# Microbial genetic composition regulates host social behavior

**DOI:** 10.1080/19490976.2025.2536091

**Published:** 2025-07-23

**Authors:** Ruijie Bai, Tao Wang, Rongrong Gu, Yawei Cai, Juntao Chen, Wen Cai, Dianshuang Zhou, Ying Li, Jixun Luo, Xiangming Wang, Zuobin Zhu

**Affiliations:** a Jiangsu Engineering Center for Precision Diagnosis and Treatment Research of Polygenic Diseases, Key Laboratory of Genetic Foundation and Clinical Application, Department of Genetics, Xuzhou Medical University, Xuzhou, China; b The First Clinical College, Xuzhou Medical University, Xuzhou, China; c Medical Technology College, Xuzhou Medical University, Xuzhou, China; d School of Life Sciences, Huzhou University, Huzhou, Zhejiang, China; e Department of Cell Biology, School of Basic Medical Sciences, Laboratory for Clinical Medicine, Capital Medical University, Beijing, China

**Keywords:** *Caenorhabditis elegans*, gut microbiota, social behavior, genetic variation, tyrosine metabolism

## Abstract

The co-evolutionary relationship between gut microbiota and their hosts is influenced by microbial genetic variation, which enables adaptation to host environmental changes, modifies metabolic processes, and refines host–microbiota interactions. Investigating how gut microbial genetic variations influence host neurobehavior can provide insights into the pathogenesis of neurological disorders. In this study, we screened a comprehensive single-gene knockout library of *Escherichia coli* (*E. coli*) and identified 370 mutant strains that reduced social behavior in *Caenorhabditis elegans* (*C. elegans*). Notably, five mutations in the L-tyrosine biosynthesis pathway significantly alter the social aggregation behavior of *C. elegans* via the TGF-β signaling pathway. These findings highlight the importance of considering both microbial genetic variation and community composition in the examination of gut microbe-host neurobehavioral interactions. The establishment of this relationship provides a reference and experimental basis for the development of genetically engineered probiotics aimed at regulating host behavior.

## Introduction

Gut microbiota plays a crucial role in regulating host neurobehavior through the gut-brain axis. Metabolites from gut microbiota, such as short-chain fatty acids (SCFAs), transmit signals along this axis and show therapeutic potential in neurodegenerative diseases by increasing dopaminergic neurons and reducing glial cell activation.^1^ Variations in microbial metabolites, influenced by genetic diversity, can alter metabolic pathways, changing the types and quantities of metabolites that affect brain function.

Advancements in metagenomics have improved our understanding of the gut microbiome–host relationship.^2^ However, current omics technologies are still limited in investigating specific microbial genetic variations and their effects on host functions. This limitation is, in part, attributable to the intricate nature of mammalian gut microbiota and the difficulties associated with the preparation of germ-free animal models. The gut-microbiota-brain axis shows promise as a therapeutic target for central nervous system disorders. Recent studies indicate that fecal microbiota transplantation (FMT) can ameliorate certain behavioral symptoms in children with autism spectrum disorder (ASD).^3^ However, FMT’s clinical application is hindered by the complexity of and lack of standardized protocols, leading to inconsistent treatment outcomes.^3^

The coevolutionary relationship between gut microbes and their hosts is shaped by microbial genetic variation, enabling them to adapt to fluctuations in the host environment, modulate metabolic functions, and refine interactions with the host.^4–6^ Despite the high complexity and relatively low mutation rate of microbial genomes, which limit the capacity for most microbial genes to undergo substantial genetic changes over short timescales, understanding how microbial genetic variation influences host social behavior remains crucial for elucidating the mechanisms underlying social behavioral disorders. To tackle this challenge, we selected single-gene mutant *E. coli* as a model organism to systematically explore how specific genetic variants impact host social behaviors and the molecular pathways through which these variants influence host behavioral phenotypes.

In this study, *C. elegans* was used to investigate the relationship between intestinal microbial genetic variation and host behavior. This model provides advantages such as a well-characterized genetic background, controlled germ-free conditions, and colonization by a single bacterial strain,^7^ enabling precise studies of microbial-host interactions. The social behavior of *C. elegans* is linked to the nervous system,^8^ and its short life cycle and rapid reproductive capabilities render it particularly suitable for high-throughput screening of the relationships between bacterial genetic variations and host phenotypes. We aim to elucidate how key microbial genes regulate host social behaviors and provide a foundation for developing genetically engineered probiotics.

## Results

### *Microbial genetic variation influences social behaviors of* C. elegans *by modulating metabolic pathways*

**Figure 1.**
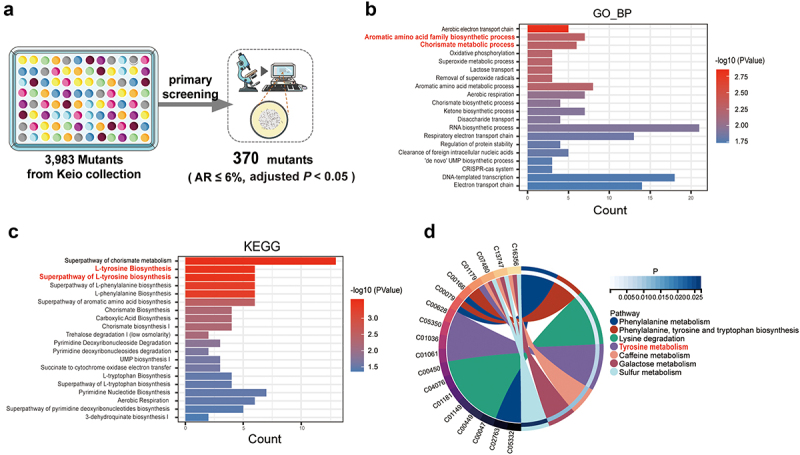
(a) Schematic representation of the screening process for single-gene knockout *E. coli* mutants that reduce the social behavior of *C. elegans*. (b) GO analysis of the genes in *E. coli* exhibiting reduced social behavior. (c) KEGG pathway analysis of the genes in *E. coli* exhibiting reduced social behavior. (d) Metabolic annotation analysis of genes in *E. coli* associated with reduced social behavior.

*C. elegans* exhibits basic social aggregation behaviors under natural conditions, and changes in these behaviors serve as a reliable indicator of alterations in the nervous system. In this study, we screened a genome-wide single-gene knockout library of *E. coli* in N2 *C. elegans* to identify genetic factors reducing aggregation. From 3,983 *E. coli* knockout strains,^9^ 370 mutants exhibited a significant reduction in the aggregation behavior of *C. elegans* (Figure 1(a), Table S1). GO and KEGG analyses showed that the decrease of tyrosine synthesis in *E. coli* was crucial for reduced worm aggregation behavior (Figure 1(b,c), Table S2). We further investigated the association between metabolites produced by 3,983 *E. coli* mutants and the social aggregation behavior of *C. elegans*, discovering that tyrosine was significantly enriched among the differentially abundant metabolites (Figure 1d). These findings suggest that decreased tyrosine synthesis due to microbial genetic variation is a key mechanism by which *E. coli* influences worm social behavior.

### *Gut bacterial tyrosine synthesis gene influences* C. elegans *social behavior*

**Figure 2.**
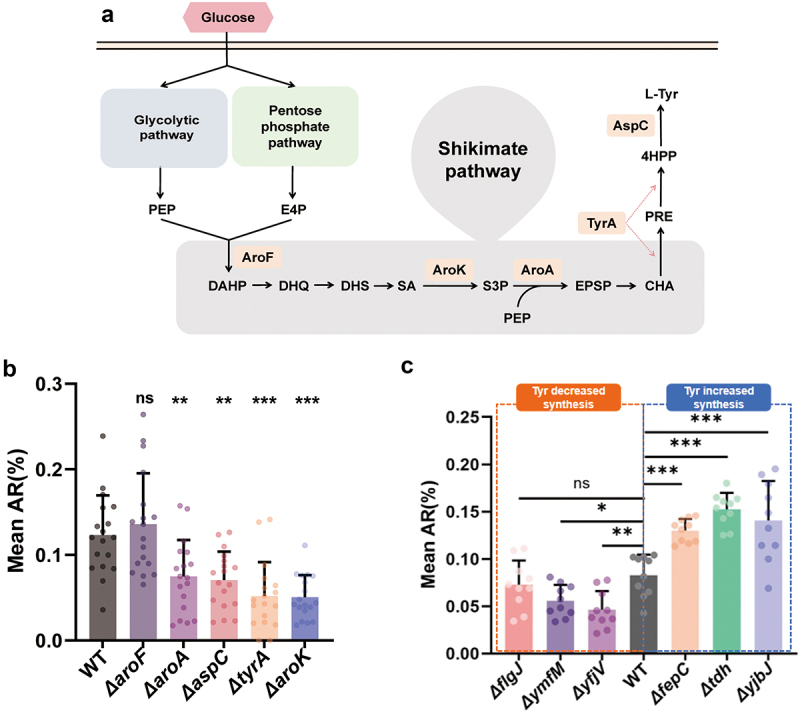
(a) Schematic illustration of tyrosine synthesis via the shikimate pathway. (b) The aggregation rates of *C. elegans* fed with wild-type, Δ*aroF*, Δ*aroA*, Δ*aspC*, Δ*tyrA*, and Δ*aroK E. coli*, analyzed using one-way ANOVA (*n* = 18). (c) The aggregation rates of *C. elegans* that were fed mutant strains, which either decreased (Δ*flgJ*, Δ*ymfM*, Δ*yfjV*) or increased (Δ*fepC*, Δ*tdh*, Δ*yjbJ*) tyrosine production in comparison to wild-type *E. coli*, were examined using one-way ANOVA (*n* = 10). Bar graphs represent the mean ± SD. *p < 0.05, **p < 0.01, ***p < 0.001. “Δ” denotes gene knockout.

The key metabolic pathway for tyrosine synthesis in *E. coli* is the shikimate pathway, involving a series of enzymatic reactions. Key enzymes include DAHP synthase (*aroF*), shikimate kinase (*aroK*), 5-enolpyruvoyl shikimate-3-phosphate synthetase (*aroA*), chorismate mutase, prephenate dehydrogenase (*tyrA*), and aspartate aminotransferase (*aspC*)^10^(Figure 2(a)). *C. elegans* fed with bacteria carrying *tyrA*, *aspC*, *aroK*, and *aroA* mutants exhibited significantly reduced social behavior (Figure 2(b)), supporting the hypothesis that decreased levels of tyrosine are associated with diminished social aggregation. Accordingly, we anticipate that mutants exhibiting low expression of tyrosine will demonstrate a reduction in social aggregation, whereas mutants with elevated tyrosine expression will exhibit an increase in social aggregation. As anticipated, two further mutants exhibiting low tyrosine expression, namely *ymfM* and *yfjV*, as indicated by metabolomic data,^11^ demonstrated a decrease in worm social aggregation. Conversely, feeding *C. elegans* with high-tyrosine-expression mutants (*tdh*, *fepC*, and *yjbJ*) increased their social aggregation (Figure 2(c)).

### *Tyrosine serves as a critical regulatory amino acid influencing social behavior in* C. elegans

**Figure 3.**
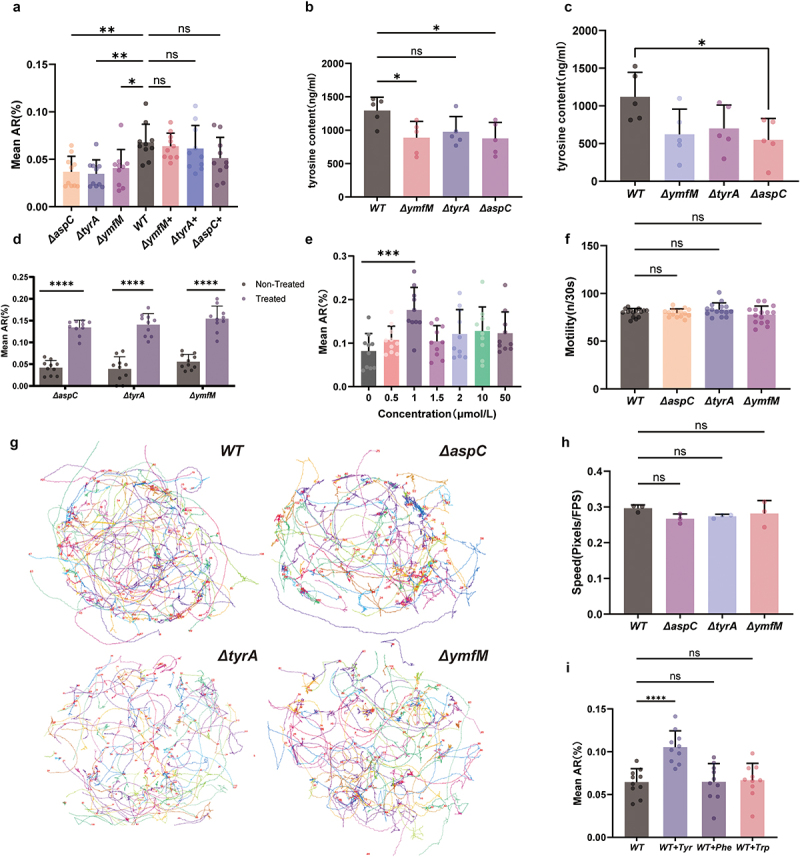
(a) the aggregation rates of *C. elegans* fed with low-tyrosine expression mutants (Δ*ymfM*) and tyrosine synthesis-deficient mutants (Δ*aspC*, Δ*tyrA*), as well as their corresponding gene-complemented strains (Δ*ymfM*+, Δ*aspC*+, Δ*tyrA*+), analyzed using one-way ANOVA (*n* = 10). (b) Tyrosine levels were quantified by chromatography-mass spectrometry in *E. coli* knockout strains (*aspC*, *tyrA*, and *ymfM*), analyzed using one-way ANOVA (*n* = 5). (c) Tyrosine levels in the *C. elegans* were quantified by chromatography-mass spectrometry after feeding with the wild-type BW25113 *E. coli* and knockout strains (Δ*aspC*, Δ*tyrA*, and Δ*ymfM*), analyzed using one-way ANOVA (*n* = 5). (d) The aggregation rates of *C. elegans* fed with *E. coli* mutant strains Δ*aspC*, Δ*tyrA*, Δ*ymfM* treated with tyrosine or not, evaluated using Student’s t-test (*n* = 10). (e) The aggregation rates of *C. elegans* fed with different concentrations of tyrosine，analyzed using one-way ANOVA (*n* = 10). (F) the head swinging speed of *C. elegans* in liquid medium. analyzed using one-way ANOVA (*n* = 15). (g) Movement trajectories of *C. elegans* on 35-mm NGM solid medium over a 2-minute period, and (h) Quantification of movement speed. Approximately 100 worms each petri plate were analyzed, with movement speed quantified in pixels per frame, analyzed using one-way ANOVA (*n* = 3). (i) The aggregation rates of *C. elegans* fed with wild-type BW25113 *E. coli* supplemented with tyrosine, phenylalanine, and tryptophan，analyzed using one-way ANOVA (*n* = 10). Bar graphs represent the mean ± SD. *p < 0.05, **p < 0.01, ***p < 0.001, ****p < 0.0001. “Δ” denotes gene knockout. “+” denotes gene complementation.

To further verify that *E. coli* knockout strains directly influence *C. elegans* behavior through these specific gene mutations rather than via other physiological processes of *C. elegans*, complementation experiments were conducted using *aspC*, *tyrA*, and *ymfM* knockout *E. coli* strains. The results demonstrated that expressing the normal *aspC* and *tyrA* genes in the *E. coli* mutants that are deficient in *aspC* and *tyrA*, respectively, rescued the negative impact on social aggregation (Figure 3(a)). Additionally, the aggregation behavior of *C. elegans* was significantly restored after the normal gene complementation of the tyrosine low-expression mutant strain (*ymfM*) (Figure 3(a)).

Above results suggest that tyrosine is an essential molecule for the aggregation behavior of *C. elegans*. To verify this hypothesis, tyrosine levels were quantified by chromatography-mass spectrometry in *E. coli* knockout strains (*aspC*, *tyrA*, and *ymfM*) as well as in worms fed with these strains. The results demonstrated that the *aspC*, *tyrA*, and *ymfM* knockout strains exhibited reduced tyrosine level (Figure 3(b)), which consequently led to a relatively lower tyrosine content in the *C. elegans* (Figure 3(c)). In addition, the addition of tyrosine to the culture medium of worms fed with *aspC*, *tyrA*, and *ymfM* knockout *E. coli* strains restored their social behavior (Figure 3(d)). In contrast, supplementation of tyrosine in the wild-type BW25113 strain further enhanced social aggregation behavior. As the concentration of tyrosine increased, the social behavior of worms gradually improved, reaching its peak effect at a concentration of 1 μmol/L. Beyond this concentration, the impact on *C. elegans* social behavior diminished (Figure 3(e)). As variations in movement ability may potentially influence the social aggregation behavior of *C. elegans*, we examined the movement ability of *C. elegans* exposed to *aspC*, *tyrA*, *ymfM* mutant *E. coli* and wild-type BW25113 *E. coli* in liquid medium, and found that the mutant bacteria did not affect the *C. elegans* head swinging speed (Figure 3(f)). Furthermore, we evaluated their movement capabilities, including movement trajectory and movement speed, after feeding with *aspC*, *tyrA*, and *ymfM* mutant *E. coli* strains on solid NGM medium. Our results indicated that no significant differences were observed in these parameters (Figure 3(g,h)).

Given that tyrosine is an aromatic amino acid, we also investigated the potential effects of other aromatic amino acids, such as tryptophan and phenylalanine, on *C. elegans* aggregation behavior. To this end, we created 1 μmol/L solutions of tyrosine, phenylalanine, and tryptophan to perform the behavior experiments. The results demonstrated that tyrosine significantly promoted *C. elegans* aggregation in comparison to the control without amino acid supplementation, whereas neither phenylalanine nor tryptophan exhibited a similar effect (Figure 3(i)). Thus, phenylalanine and tryptophan did not substantially influence worm aggregation behavior. Collectively, these findings underscore that microbial genes directly modulate *C. elegans* behavior, with tyrosine metabolism representing a key regulatory pathway.

### Microbial metabolite tyrosine influences the host’s TGF-β signaling pathway

**Figure 4.**
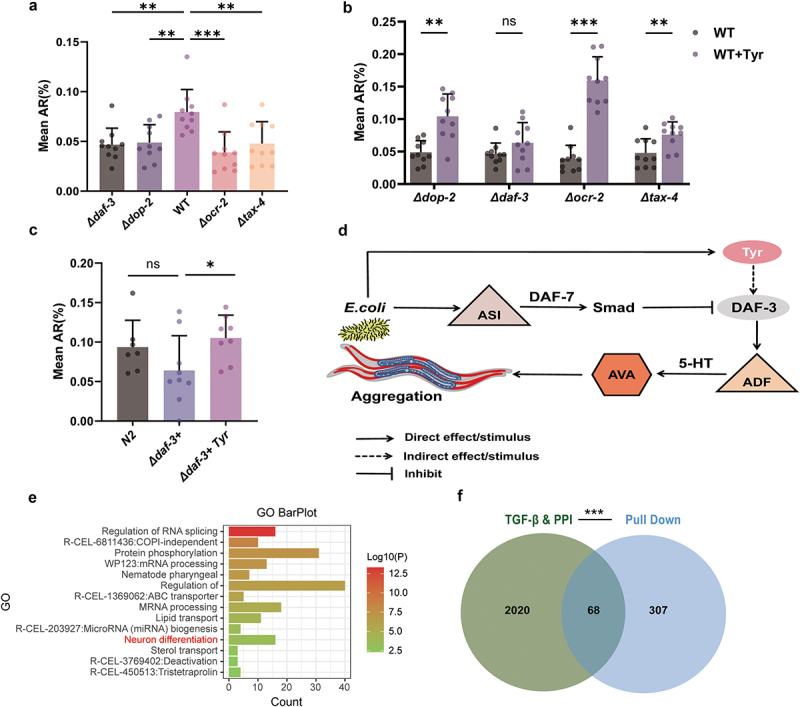
(a) The aggregation rates of *tax-4*, *ocr-2*, *dop-2*, *daf-3* mutants and wild-type N2 *C. elegans* feeding with wild-type *E. coli*, analyzed using one-way ANOVA (*n* = 10). (b) Tyrosine rescue experiments in *tax-4*, *ocr-2*, *dop-2*, *daf-3* mutant strains of *C. elegans*, evaluated using Student’s t-test (*n* = 10). (c) The aggregation rates of the N2 *C. elegans*, *daf-3* mutant gene-complemented strain (*daf-3*+), and *daf-3* mutant after genetic complementation and tyrosine supplementation were examined while they were fed wild-type *E. coli*, utilizing one-way ANOVA for the analysis (*n* = 8). (d) Schematic representation of tyrosine’s interaction with the TGF-β/DAF-7 signaling pathway. (E) GO functional analysis of proteins interacting with tyrosine. (f) Venn diagram illustrating the overlap between tyrosine-interacting proteins, the TGF-β signaling pathway, and interacting proteins within this pathway. Bar graphs represent the mean ± SD. *p < 0.05, **p < 0.01, ***p < 0.001. “Δ” denotes gene knockout. “+” denotes gene complementation.

The *tax-4*,^12^ TRPV signaling pathway,^13^ *daf-7* transforming growth factor-β (TGF-β) signaling pathway,^14^ and the dopaminergic signaling pathway^15^ are well established regulators of social behavior in both *C. elegans* and mice. To explore the regulatory mechanisms by which tyrosine enhances social behavior in *C. elegans*, we examined the aggregation behavior in mutants of *tax-4.* The GCY-35/TAX-4 signaling pathway12, TRPV signaling pathway13, DAF-7/transforming growth factor-β (TGF-β) signaling pathway14, and the dopaminergic signaling pathway15 are well-established regulators of social behavior in both C. elegans and mice. We observed that the aggregation behavior of *tax-4*, *ocr-2*, *daf-3*, and *dop-2* mutants was significantly diminished compared to the wild-type N2 strain (Figure 4(a)). Dietary supplementation with tyrosine enhanced the social aggregation behavior in *tax-4*, *ocr-2*, and *dop-2* mutants. However, tyrosine did not alter the aggregation behavior in *daf-3* mutants (Figure 4(b)).

To further confirm the critical role of TGF-β signaling in regulating *C. elegans* social behaviors, we conducted genetic complementation experiments in *daf-3* mutants. The results demonstrated that *daf-3* mutants with genetic complementation restored their aggregation behavior to a level comparable to that of wild-type N2 *C. elegans* (Figure 4(c)). We also supplemented tyrosine to the *daf-3* mutant that had undergone genetic complementation. The results showed that the aggregation behavior was significantly enhanced (Figure 4(c)). These findings suggest that tyrosine promotes aggregation behavior through the TGF-β/DAF-7 signaling pathway in *C. elegans*.

In *C. elegans*, DAF-7 signaling from ASI sensory neurons inhibits aggregation behavior by suppressing *tph-1* gene expression in ADF neurons. This suppression reduces 5-HT synthesis, which is critical for aggregation.^16,17^ DAF-7 achieves this by activating SMAD proteins (e.g., DAF-8 and DAF-14) via its receptors (DAF-1 and DAF-4), thereby inhibiting DAF-3 activity^18^ (Figure 4(d)). Additionally, 375 tyrosine-interacting proteins were identified by pull-down assay in *C. elegans*, with significant enrichment of proteins involved in neuron differentiation and the TGF-β signaling pathway (Figure 4(e,f)).

## Discussion

In this study, *C. elegans* was utilized as a model organism to elucidate the intrinsic relationship between intestinal microbial genetic variation and host social behavior regulation. Through comprehensive and systematic screening of single-gene knockout strains of *E. coli*, we successfully identified a series of bacterial gene mutations that significantly reduce aggregation behavior of *C. elegans*. To strengthen the causal link between microbial genetic variations and host behavior, we performed bacterial genetic complementation experiments for key genes (*tyrA*, *aspC*, *aroK*). As shown in Figure 3(a), expression of these genes in mutant *E. coli* strains rescued aggregation behavior in *C. elegans*, confirming their direct role in modulating social behavior. These findings provide robust evidence that microbial genetic variation, rather than generalized metabolic defects, drives behavioral changes in the host.

Another key finding of this study was the identification of microbially derived tyrosine as a critical metabolite influencing *C. elegans* aggregation behavior. Tyrosine levels were significantly elevated in worms fed tyrosine-producing *E. coli* strains compared to those fed knockout strains. Furthermore, dose–response experiments revealed a non-linear relationship between tyrosine supplementation (0.1–50 μmol/L) and aggregation behavior (Figure 3(e)), with 1 μmol/L showing maximal effect. These data reinforce the hypothesis that microbially derived tyrosine may play a key role in regulating worm social behavior.

Mechanistic studies demonstrated that tyrosine exerts its effects by activating the TGF-β/DAF-7 signaling pathway in *C. elegans*. In *tax-4*, *ocr-2*, and *dop-2* mutants, tyrosine supplementation partially restored aggregation behavior, whereas this restoration was not observed in *daf-3* mutants (a core SMAD protein in the TGF-β pathway) (Figure 4(b)). Upon restoration of *daf-3* function in *C. elegans*, tyrosine supplementation significantly enhanced social aggregation (Figure 4(c)). Pull-down assays demonstrated that tyrosine may directly or indirectly interact with components of the TGF-β pathway, such as DAF-1 and DAF-4, as well as proteins associated with neuronal differentiation (Figure 4(e,f)). These findings further support the potential link between tyrosine and the TGF-β signaling pathway. This observation highlights similarities among different species, as TGF-β is known to regulate neuroinflammation and synaptic plasticity in mammals,^19,20^ suggesting that microbiota-derived metabolites might influence higher cognitive functions via conserved pathways.

It is worth noting that the role of microbiota in neurobehavioral regulation demonstrates cross-species complexity. While this study employs *C. elegans* as a model organism, microbial genetic variations have also been confirmed to regulate neurobehavioral phenotypes such as locomotion and sleep in other non-mammalian models, including *Drosophila melanogaster*.^21^ In mammals, growing evidence supports the notion that gut microbiota influences neurobehavior via the gut-brain axis, affecting processes such as neuroinflammation regulation and the progression of neurodegenerative diseases.^22,23^

In particular, the three aromatic amino acids-phenylalanine, tyrosine, and tryptophan-play a pivotal role in the gut-brain axis. As essential metabolites produced by gut microbiota serve not only as precursors for various secondary metabolites but also directly contribute to neurotransmitter synthesis (e.g., tyrosine acts as a precursor of dopamine), thus influencing brain health and behavioral regulation.^24^ Existing studies indicate that the composition of the gut microbiome modulates host social decision-making, with this effect linked to alterations in fasting serum levels of the dopamine precursor tyrosine.^25^ A systematic review reveals that reduced plasma tyrosine levels correlate with autism spectrum disorder,^26^ while L-tyrosine supplementation effectively alleviates behavioral abnormalities in socially isolated mice^27^ and has been shown to mitigate autism-like behaviors in mice by reshaping the gut microbiota.^28^ These findings align with the conclusion of this study: “tyrosine regulates social behavior through conserved signaling pathways” across species, suggesting that microbiota-derived tyrosine may influence neurobehavior in higher animals via analogous mechanisms. Nevertheless, considering interspecies differences, whether TGF-β directly regulates social behaviors in mammals remains an unresolved question warranting further exploration.

Neurobehavioral experiments are inherently susceptible to both objective and subjective variables. To minimize potential confounding factors, our experimental design incorporated rigorous controls to standardize critical environmental parameters. Specifically, the temperature was precisely maintained at (20 ± 0.5°C) using a precision-controlled incubator. Bacterial density was adjusted to an optical density (OD600 = 0.6) through spectrophotometry, and food availability was strictly regulated to ensure consistency across all replicates. To further enhance reliability, social behaviors associated with tyrosine metabolism, such as those observed in *E. coli* mutants and tyrosine-supplemented *C. elegans*, were systematically analyzed using automated quantification tools to reduce subjective bias.^29^

In summary, this work enhances our understanding of how microbial genetic variation influences host behavior through specific metabolic pathways. Although the translational implications for social behavior disorders are promising, further validation in mammalian systems is essential to translate these findings into clinical applications. Our comprehensive method, which merges genetic screening, metabolomics, and neural tracing, provides a strong foundation for thoroughly analyzing the interactions between microbiota and their hosts. However, the feasibility of applying this single-bacterium intervention strategy within the context of complex microbiota remains unclear. Specifically, whether microbiota interactions modulate the metabolic output of a single strain via metabolic networks, such as branched-chain amino acid (BCAA) competition, warrants further investigation.

## Methods

*C. elegans* strains, including N2 (Bristol wild-type), PR678 (*tax-4* knockout), C×4544(*ocr-2* knockout), L×702(*dop-2* knockout), and CB1376 (*daf-3* knockout), were cultured at 20°C on standard NGM agar plates with pre-cultured bacteria, following established protocols.^30^ All strains were obtained from the Caenorhabditis Genetics Center (CGC). The knockout *E. coli* with low expression of *aspC*, *tyrA*, and *ymfM* mediated by the pEC19 plasmid (the gene complementation strain for *aspC*, *tyrA*, and *ymfM* mutants) was provided by Hangzhou Fenghai Biotechnology Co., Ltd. The wild type *daf-3* genomic fragment, which is 9127 base pairs long and includes the *daf-3* promoter, gene, and 3’ UTR, was amplified with LA Taq. The primers used for amplification were as follows: forward primer – gacggtttcaaggctaagatc, and reverse primer – ggaccagtgactgatgacta. This *daf-3* genomic fragment was then injected into *daf-3* mutant, using P*myo-2*::mCherry as a co-injection marker.

### *E. coli deletion collection screen for changes in* C. elegans *aggregation behavior*

The screening experiment was carried out as in previous studies.^31^

#### Strain preparation

The Keio Collection of 3,983 *E. coli* single-gene deletion strains was cultured in an LB medium supplemented with 25 μM kanamycin. Overnight incubations (12–14 h, 37°C) preceded density normalization to OD600 = 0.6. Post-cultivation, cells were pelleted (6,000 rpm, 5 min) and resuspended in an antibiotic-free LB medium to generate standardized suspensions.

#### Plate assembly

About 150 μL aliquots of bacterial culture were pipetted onto 60 mm Petri dishes, forming uniform lawns after 12–24 h air-drying in a laminar flow hood. This procedure ensured consistent bacterial distribution for subsequent behavioral assays.

#### C. elegans *aggregation assay*

*C. elegans* N2 larvae were synchronized at the L1 stage using M9 buffer overnight. Approximately 300 L1 larvae were then transferred to plates seeded with *E. coli* mutants and cultured for 48 h. Subsequently, the larvae were redistributed onto fresh plates (approximately 100 worms per plate) and grown to adulthood for an additional 10 h. Aggregation behavior was imaged using a Motic SMZ-171 stereomicroscope. The images of *C. elegans* aggregation were analyzed using WormTrack software.^29^ The principle of the software analysis is as follows: Aggregation occurred when the body contact area between two worms was 50% or more, or when their bodies intersected.^32,33^ For each plate, both the total number of worms (N_im_) and the number of worms aggregated at the edge of the bacterial lawn (N_ij_) were counted. The Edge Aggregation Rate (M) was calculated using the formula: *M*=$\sum_{i=1}^{n}\mathrm{ai}(\frac{\mathrm{Nij}}{\mathrm{Nim}})/n$.

### Quality control measures

Neurobehavioral experiments are inherently susceptible to both objective and subjective variables. To minimize potential confounding factors, all experimental parameters (temperature, bacterial density, worm density) were monitored using calibrated instruments with documented measurement uncertainties. Blinded image analysis protocols prevented experimenter bias during behavioral quantification. Specifically, the temperature was precisely maintained at (20 ± 0.5°C) using a precision-controlled incubator, the bacterial density was adjusted to an optical density (OD600 = 0.6) through spectrophotometry, and food availability was strictly regulated to ensure consistency across all replicates.

#### Prediction of tyrosine–protein interactions

In this study, tyrosine–protein interactions in adult *C. elegans* tissues were investigated using biotin labeling and molecular docking. Tissues were labeled with acetyl eugenol, and proteins were extracted and conjugated. Conjugates were purified with streptavidin-coated beads to remove nonspecific binding. Specific interactions were analyzed. Docking studies were conducted using AutoDock and Vina, with receptor proteins from PDB and acetyl eugenol structure from PubChem. Key binding regions were visualized in PyMOL. This approach integrated biotin labeling, affinity purification, and computational docking.

#### Measurement of tyrosine

Bacterial cultures with an OD600 of 0.6 and 1000–2000 worms were lysed using an ultrasonic disruptor, and the lysate was mixed with a methanol solution containing a specific ratio of methanol, acetonitrile, and an internal standard. After vortexing and ice-cold ultrasonic extraction, the samples were incubated, centrifuged, and the supernatant was collected, dried, and reconstituted in an acetonitrile-water solution. Subsequently, tyrosine detection was performed using a Thermo DGLC dual ternary UHPLC system with a Waters HSS T3 column for separation. Both reverse-phase and normal-phase conditions were employed, and mass spectrometry analysis was conducted using a Q Exactive high-resolution mass spectrometry system with specific parameters set for optimal detection.

#### Preparation of tyrosine culture medium

To prepare a 3 mmol/L tyrosine standard solution, dissolving 0.01087 g of tyrosine in 20 mL deionized water at 37°C for 2 h. For a 1 μmol/L tyrosine NGM culture medium, adding 16.67 μL of the standard solution to 50 mL sterile NGM medium. Mix thoroughly, pour into petri dishes, let solidify, and store upside down.

#### Measurement of locomotion ability

*C. elegans* exhibits social aggregation behavior when cultured on solid NGM medium. A video was recorded using a Motic SMZ-171 stereomicroscope equipped with a camera. Given the complex movement trajectories of worms on solid NGM medium, it is challenging to directly measure their actual movement distance. However, the dynamic movement speed of *C. elegans* can be quantified as pixels per frame. WormTrack software employs this principle to analyze worm movement speed. In this study, WormTrack was used to analyze the movement trajectories of approximately 100 worms within the 2-min recording period.^29^

#### Statistical analysis

Significant differences in metabolites of mutant bacteria were identified using t-tests on data from this study and public platforms.^11^ Functional enrichment analysis was then conducted using Metaboanalyst 6.0 for metabolites with significant differences.^34^ In this study, a two-layer validation strategy was implemented to minimize false positive results. First, over 30 independent replicate experiments were conducted under standardized conditions (20 ± 0.5°C, OD600 = 0.6) to establish a baseline aggregation rate for wild-type *E. coli* (>6%). Subsequently, rigorous statistical filtering was applied to screen 3,983 mutant strains using Student’s t-test with Bonferroni correction (adjusted *p* < 0.05). A total of 370 mutant strains exhibiting significantly lower aggregation rates than the wild type (<6%) were identified and selected. In the mechanistic analysis, two-tailed Student’s t-tests were employed for two-group comparisons, while the one-way ANOVA followed by Tukey’s post hoc test was used for multi-group comparisons. All analyses were conducted using R 4.3.1 and GraphPad Prism 10.

## Supplementary Material

Table S1.xlsx

Table S2.xlsx
